# Progress in human liver organoids

**DOI:** 10.1093/jmcb/mjaa013

**Published:** 2020-04-01

**Authors:** Lulu Sun, Lijian Hui

**Affiliations:** 1 State Key Laboratory of Cell Biology, Chinese Academy of Sciences Center for Excellence in Molecular Cell Science, Shanghai Institute of Biochemistry and Cell Biology, Chinese Academy of Sciences, University of Chinese Academy of Sciences, Shanghai 200031, China; 2 Bio-Research Innovation Center, Shanghai Institute of Biochemistry and Cell Biology, Chinese Academy of Sciences, Suzhou 215121, China; 3 Institute for Stem Cell and Regeneration, Chinese Academy of Sciences, Beijing 100101, China; 4 School of Life Science and Technology, ShanghaiTech University, Shanghai 201210, China

**Keywords:** liver organoid, progress, perspectives

## Abstract

Understanding the development, regeneration, and disorders of the liver is the major goal in liver biology. Current mechanistic knowledge of human livers has been largely derived from mouse models and cell lines, which fall short in recapitulating the features of human liver cells or the structures and functions of human livers. Organoids as an *in vitro* system hold the promise to generate organ-like tissues in a dish. Recent advances in human liver organoids also facilitate the understanding of the biology and diseases in this complex organ. Here we review the progress in human liver organoids, mainly focusing on the methods to generate liver organoids, their applications, and possible future directions.

## Organoids in general

Organoids are now used to term the miniatured organ-like cultivates in 3D cultures (Lancaster and Knoblich, 2014). Different from cells in 2D cultures, organoids are derived by self-organization of groups of cells, including stem cells, adult tissue progenitors, and diverse types of mature cells from an organ, in a spatial pattern similar to the counterpart tissues *in vivo*. Back to the last centuries, scientists have demonstrated the potential of dissociated cells to re-establish a tissue-like association *in vitro* (Moscona and Moscona, 1952; Weiss and Taylor, 1960). However, the booming of this new field has to wait until recent decades the advances in the understanding of morphogenesis, the development of stem cell technologies, and the preparation of proper extracellular matrices (ECMs) (Simian and Bissell, 2017). As a fact, during the last 10 years, a large number of studies have reported the generation of organoids of diverse organs, such as the brain, retina, kidney, lung, gut, and liver (Willyard, 2015).

Organoids are an easily accessible *in vitro* model that recapitulates certain structures and functions of their counterpart organs *in vivo,* which are not observable in individual cells or 2D-cultured cell sheets, and, as such, greatly expand our research scopes. It is interesting that organoids not only introduce the structural and functional changes to the cell but also bring other underappreciated features of the tissue. For example, it has been shown unexpectedly that stabilized tissue architectures could support the improved fidelity of chromosomes, likely through an integrin-mediated establishment of cell polarity (Knouse et al., 2018). Organoids have been proven as a powerful tool to study the development and regeneration as well as a potential model to understand human disorders and explore disease treatment (Clevers, 2016). Brain organoids derived by differentiation from pluripotent stem cells (PSCs) were applied to model cerebral cortex development, brain evolution, and nervous disorders, including autism, Alzheimer’s disease, and Zika virus (ZIKV) infection (Qian et al., 2019). Intestinal organoids generated from single Lgr5^+^ stem cells were found to build crypt–villus structures with a stratified epithelium, presenting major types of cells in the gut (Sato et al., 2009), and such intestinal organoids have been used in mimicking tissue regeneration and cancer development (Nakamura and Sato, 2018).

Beyond that, organoids provide new tools for translational research and alternative organ replacement therapies in clinics. Cancer organoids generated by genetic engineering of normal cells or from primary tumor tissues hold the promise for drug development and personalized cancer treatment (Tuveson and Clevers, 2019). Engineered organoids by sequential introduction of oncogenic mutations have been used in modeling the initiation and developmental process of diverse types of cancers, such as ductal pancreatic cancers (Huang et al., 2015) and colorectal cancers (Crespo et al., 2017). Moreover, biobanks of patient-derived organoids (PDOs) from prostate cancers (Gao et al., 2014), breast cancers (Sachs et al., 2018), colorectal cancers (van de Wetering et al., 2015)*,* ovarian cancers (Kopper et al., 2019), and bladder cancers (Lee et al., 2018) have been generated as invaluable resources for precision medicine. In addition, pilot studies have demonstrated the application of organoids in regenerative medicine. For example, colon (Yui et al., 2012) and liver organoids (Huch et al., 2013; Takebe et al., 2013) differentiated from stem cells showed some therapeutic effects in mice.

With the fast development and wide demands of organoid technologies, challenges have been posed on traditional 3D cultures in order to achieve the precise control of organoid formation and to realize the complex microenvironment of a given organ. As a consequence, it opens up new research frontiers in the reproducibility of the organoid culture, the inclusion of cells from other functional lineages, and the alliance with gene editing for acquisition of complex organoids (Park et al., 2019). As a hallmark of this trend, the development of individual brain organoids containing diverse cell types of human cerebral cortex has been achieved by adaptation and optimization of several protocols (Velasco et al., 2019). Other researchers are dedicated to generate complex organoids to better mimic the disease status *in vivo*, including engineering vascular systems (Cakir et al., 2019; Homan et al., 2019; Wimmer et al., 2019), addition of nervous system (Workman et al., 2017), and assembling different parts of the tissue together (Harrison et al., 2017).

The liver as the biggest visceral organ is essential for many important life-sustaining functions, such as carbohydrate and lipid metabolism, drug detoxification, bile secretion, and plasma protein production. It has attracted great interests in developing liver organoids in the last years. Liver organoids thus generated provide structural and functional advantages for understanding liver development and the onset of liver diseases *in vitro*. Here, we summarize the recent progress in generation of liver organoids and their potential applications.

## The structure of the liver

**Figure 1:**
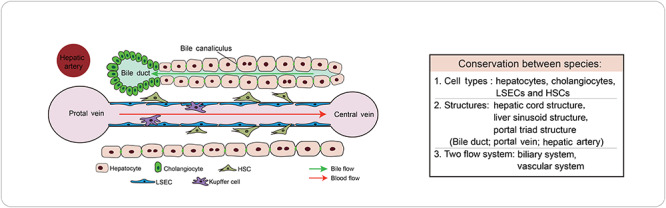
Schematic diagram of the liver lobule and the conservation between species. Liver lobule, containing two flow systems, biliary system and vascular system, is a fundamental and conserved structure of the liver between species. Oxygenous blood and nutrition flow into the liver from the portal vein and hepatic artery at the periportal area to the central veins at the pericentral area. The bile secreted from hepatocytes is collected into the bile duct, which flows in opposite directions. The portal vein, hepatic artery, and bile duct make up the portal triad structure. Plates of hepatocytes line along the liver sinusoidal capillaries in a radial distribution from the periportal area to the pericentral area. LSECs, Kupffer cells, and other resident macrophages are located at the luminal side of the sinusoids, whereas HSCs are positioned outward.

The liver consists of thousands of basic structural and functional units, called liver lobules, which are evolutionally conservative between species in vertebrates (Si-Tayeb et al., 2010a; Miyajima et al., 2014; Shiojiri et al., 2018). The liver lobule is constituted with parenchymal cells, mainly hepatocytes, and nonparenchymal cells, including cholangiocytes, liver sinusoidal endothelial cells (LSECs), hepatic stellate cells (HSCs), and immune cells. Intriguingly, single-cell sequencing studies have recently proposed new types of cells in human liver lobules, which however would require functional confirmation in the future (MacParland et al., 2018; Aizarani et al., 2019). Hepatocytes and cholangiocytes are two types of epithelial cells differentiated from hepatoblasts, the embryonic liver progenitor cells, during organogenesis. Different types of liver cells are organized in particular positions along the lobule, which are essential for their own functions as well as other types of cells (Si-Tayeb et al., 2010a; Miyajima et al., 2014). Hepatocytes, playing a major role in liver functions, are arranged as cords that radiate along the hepatic sinusoids from central veins to portal veins. Hepatocytes are polarized epithelial cells with tight junctions at the lateral membranes while the basal membranes facing LSECs. The unique polarity structures are essential for hepatocyte functions. Vascular systems bring in oxygen and nutrition from portal vein and hepatic artery lined at the periportal area to the central vein, thus creating a zonation environment in the lobule. Bile canaliculi are sealed by tight junctions on the lateral membrane between neighboring hepatocytes, functioning to collect the bile secreted by hepatocytes. The bile secreted from bile canaliculi flows in opposite directions of blood and is excreted out of the liver from the bile duct (Figure 1).

Given such complicated structure, it is obviously difficult to generate the organized lobule structure carrying multiple liver functions *in vitro*. On the top of that, culture and expansion of primary hepatocytes (PHs) that maintained hepatic functions have been a bottleneck in the field for a long time (Bhatia et al., 2014). Only in recent years with the development of stem cell technologies, it became possible to culture and expand functional hepatocytes and cholangiocytes in the lab. Based on this progress, many exciting advances were thus emerged, ranging from culturing simple organoids that were generated from one type of cells to complex organoids assembling with diverse types of cells (Table 1).

**Table 1:** Recent advances in generating liver organoids *in vitro.*

**Category**	**Identity**	**Contents**	**ECMs or techniques**	**References**
Simple organoids	PSC differentiation	Human hepatocytes	Ultra-low cluster dishes	Ogawa et al., 2013
		Human cholangiocytes	Matrigel	Ogawa et al., 2015; Sampaziotis et al., 2015
	Transdifferentiation	Human hepatocytes	Ultra-low adherent plates	Sun et al., 2019
		Mouse hepatocytes	Ultra-low adherent plates	Yamamoto et al., 2018
	Primary cells	Fetal/adult human PHs	Matrigel	Hu et al., 2018
		Proliferated human PHs	Ultra-low adherent plates	Zhang et al., 2018; Fu et al., 2019
		Mouse Lgr5^+^ liver cells	Matrigel	Huch et al., 2013
		Human EpCAM^+^ cholangiocytes	Matrigel	Huch et al., 2015
		Mouse PHs	Matrigel	Hu et al., 2018; Peng et al., 2018
		Mouse Lgr5^+^ hepatoblasts	Matrigel	Prior et al., 2019
Complex organoids	Hepatobiliary organoid	Human PSC–hepatocytes/cholangiocytes	Matrigel	Guan et al., 2017; Wu et al., 2019
	Vascularized organoid	Human PSC–endoderm cells, PSC–endothelial cells/HUVECs, MSCs	Ultra-low adherent plates/Matrigel	Takebe et al., 2013, 2018
		Human PHs, MSCs, patterned HUVECs	Collagen and fibrin	Stevens et al., 2017

## Simple liver organoids

Hepatocytes and cholangiocytes are two major types of functional cells in the liver. Recent progress in simple liver organoids has been focusing on methods to generate organoids with either hepatocytes or cholangiocytes. Hepatocyte organoids displayed solid aggregations of well-patterned hepatocytes, accompanied with gain of hepatic polarities and improved hepatic functions. Cholangiocyte organoids often showed cystic hollow structures and performed fluid transport and cyst swelling. Here, we will describe the generation methodology and the structural and functional features of these organoids.

### Differentiation of PSCs

*In vitro* differentiation and reprogramming have been proven as successful strategies to generate functional hepatocytes and cholangiocytes in culture. Several protocols have been established to differentiate PSCs into hepatocyte-like cells and cholangiocyte-like cells following a stepwise differentiation with the addition of growth factors and chemical inhibitors in accordance with the embryonic development (Song et al., 2009; Si-Tayeb et al., 2010b; Dianat et al., 2014; Szkolnicka et al., 2014; Ogawa et al., 2015; Sampaziotis et al., 2015). The PSC-differentiated hepatocytes become further mature after assembly into 3D organoids, with striking improvement in expressions of genes associated with liver functions, such as albumin, TAT, and CYP450 genes, and mature hepatocyte surface marker ASGR1 (Ogawa et al., 2013). When PSCs were differentiated into cholangiocytes as aggregates, they presented ductal structures, with either tubular or hollow cyst morphology. Notably, they acquired apicobasal polarity with the expression of markers restricted to the apical side and the presence of primary cilia, an essential sensory organelle in controlling cholangiocyte functions (Ogawa et al., 2015; Sampaziotis et al., 2015).

### Self-assembling of reprogrammed liver cells

Besides organoid generation from PSC aggregates, self-assembling of differentiated mature cells into spheroids represents another strategy. Forced activation of liver transcriptional factors converts fibroblasts into hepatocyte-like cells directly, a process also named as transdifferentiation (Huang et al., 2011, 2014; Sekiya and Suzuki, 2011; Du et al., 2014). It was notable that hepatocytes derived from PSC differentiation or transdifferentiation showed comparable functions in many aspects (Gao et al., 2017). Transdifferentiated hepatocytes could self-organize into organoids with improved hepatocyte functions in ultra-low adherent plates (Yamamoto et al., 2018; Sun et al., 2019). Importantly, hepatocytes in the organoids were polygonal in shape, and displayed oval-shaped nuclei and bile canaliculi structures, indicating the gain of hepatic polarities. Moreover, hepatocyte organoids presented improved expressions of hepatocyte genes and functions in albumin secretion, glycogen storage, drug metabolism, and lipid transportation (Sun et al., 2019). Mechanistically, the activation of the Hippo signaling was proposed to be critical for hepatic maturation in self-assembled liver organoids. Reduced nuclear levels of Yap, which was possibly mediated by intracellular actin reorganization, facilitated the growth arrest and functional differentiation in these transdifferentiated hepatocyte organoids (Yamamoto et al., 2018).

### Self-organization of primary liver cells

Primary cells isolated from adult livers under physiological or pathological conditions also possess the ability to grow as organoids. Fetal mouse Lgr5^+^ hepatoblasts or damage-induced adult mouse Lgr5^+^ liver cells could expand as bipotent progenitor organoids *in vitr*o, which show the abilities to differentiate into functional hepatocytes (Huch et al., 2013; Prior et al., 2019). Recently, PHs isolated from mice were able to proliferate extensively *in vitro* as organoids or bipotent progenitor cells under the directions of Wnt agonists, TGFβ inhibitors, or injury-induced inflammatory cytokine TNFα by different groups (Katsuda et al., 2017; Hu et al., 2018; Peng et al., 2018). Notably, murine hepatocyte organoids could expand for ∼2–3 months *in vitro,* with induction of biliary markers and progenitor cell markers under the direction of Wnt agonists (Hu et al., 2018), whereas for TNFα-induced ones, cells could be passaged for ∼6 months without expression of those biliary or progenitor markers (Peng et al., 2018). Overall, proliferated murine hepatocyte organoids recapitulated the gene expression patterns of hepatocyte proliferation upon partial hepatectomy (PHx) *in vivo*.

Importantly, several groups have reported the culture conditions for long-term expansion of adult human hepatocytes as bi-phenotypic or liver progenitor-like cells *in vitro*. It is striking that when these expanded bi-phenotypic cells were aggregated and further cultured as organoids, they re-differentiated into mature hepatocytes with drastic improvement in hepatic functions and remarkable loss of progenitor features (Zhang et al., 2018; Fu et al., 2019). Importantly, bi-nuclei cells were detected in these organoids, which was a distinct feature of mature hepatocytes (Zhang et al., 2018). Besides adult hepatocytes, hepatocytes isolated from human fetal liver were established in organoids that maintained significant liver functions and structures for several months (Hu et al., 2018). It was also worth mentioning that primary human EpCAM^+^ ductal cells could be readily expanded as bipotent progenitor cells under defined 3D culture conditions. Genome-stable clonal cultures could even initiate from single-sorted cells, and the organoids all displayed typical duct-like phenotype (Huch et al., 2015).

## Complex liver organoids

Complex liver organoids composing multiple types of cells have been generated by several labs, with the focus on developing vascular or biliary systems. Vasculature formation by endothelial cells is fundamental for nutrition and oxygen delivery into the organ. To generate vascularized liver organoids, human endothelial cells derived from human umbilical vein endothelial cell (HUVEC) or PSC differentiation, mesenchymal stem cells (MSCs), and PSC-derived hepatic endoderm cells were cultured together in ultra-low adherent plates. These cells formed aggregates and generated human liver buds after 1 or 2 days (Takebe et al., 2013, 2017). Stromal-cell-dependent factors were involved in liver bud formation. Endothelial network was homogenously distributed in organoids. Notably, after transplantation *in vivo*, liver buds connected to the host vessels, and blood vessels became unobstructed and presented similar density and morphology to vessels in adult livers (Takebe et al., 2013). However, diverse types of cells in liver buds did not show clear sign of organized structures by histological analysis. In another study, human liver tissue seeds were constructed by engineering human PHs, endothelial cells, and stromal cells on a prefabricated pattern based on cellular signaling networks in regeneration. After transient *in vitro* culture, they were engrafted into liver failure mice, which allows for better expansion of fabricated liver organoids in response to regenerative cues when compared with randomly organized cells (Stevens et al., 2017).

For biliary system, cholangiocytes line along the biliary ducts to transport the bile secreted from hepatocytes out of the liver. Different from the vascularized organoids generated from assembling of multiple types of cells, hepatobiliary organoids were mainly generated by protocols using co-differentiation of hepatocytes and cholangiocytes from PSCs, which recapitulated several key aspects of liver organogenesis *in vivo*. Hepatocytes were tightly gathered in hepatobiliary organoids, while cholangiocytes showed cystic structures. Though both of the cells presented similar structures to hepatocytes and cholangiocytes *in vivo*, they were neither organized in a zonation pattern following liver structures *in vivo* nor performed exocrine functions (Guan et al., 2017; Wu et al., 2019). Future attempts to reinforce the structures and cellular interactions in the liver are needed for constructing complex liver organoids (Table 1).

## Application of liver organoids

**Figure 2:**
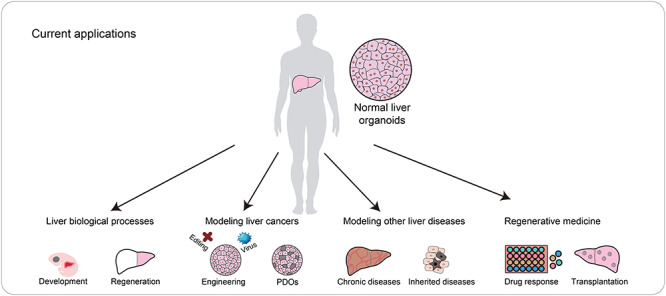
Current applications of liver organoids. Liver organoids derived from healthy donors represent ideal models in understanding the fundamental biological processes in the liver, including embryonic development and regeneration. They also show potentials in liver disease modeling. Organoids derived from diseased samples, including cancer organoids, recapitulate the original features of patients. Moreover, gene editing, virus infections, and introduction of drugs to the liver organoids are able to model the occurrence and developmental processes of diseases, including liver cancer occurrence and chronic or inherited diseases. Hopefully, healthy liver organoids could be applied in regenerative medicine. They are ideal models for screening diverse drugs and translational studies in the clinic.

The emergence of organoids enriches the toolbox in modeling embryonic development, adult tissue regeneration, and diseases and facilitates the development of future regenerative medicine as well. When specified to liver organoids, a variety of protocols have been established for their applications (Figure 2).

### Liver development and regeneration

Embryonic liver development and adult liver regeneration depend on the orchestrated interplay of multiple signaling pathways. However, the functions of these signaling pathways remain largely unresolved. PSC-derived liver organoids represent a model for studying fundamental processes during liver development. Liver organoids that are differentiated from PSCs, including hepatocyte organoids, cholangiocyte organoids, and hepatobiliary organoids, go through a stepwise differentiation and recapitulate liver development *in vivo* to some extent. Taking advantages of liver organoids, it has been revealed that paracrine signals derived from mesenchymal or endothelial cells promoted the maturation of liver organoids from PSCs, which appeared to be a model to recapitulate interactions between stromal cells and epithelial cells in liver development (Asai et al., 2017; Camp et al., 2017).

Moreover, liver organoids generated from cells with inherited mutations provide new models to analyze developmental abnormalities caused by these mutations. The Notch pathway is important for biliary tract formation, and mutations of JAG1, one of Notch ligands, cause Alagille syndrome (ALGS), an autosomal dominant genetic disorder. Indeed, liver organoids generated with cells from ALGS patients showed an impaired formation of cholangiocytes, representing a model to investigate the Notch pathway in bile duct development (Guan et al., 2017).

The liver harbors a remarkable regenerative capacity. Terminally differentiated hepatocytes and cholangiocytes are plastic to regenerate the liver under injury states, either through self-replication or lineage conversion (Li et al., 2016). Hepatocyte-dependent proliferation regenerates the lost liver mass after PHx. Interestingly, the growing murine hepatocyte organoids *in vitro* showed similar transcriptional profiles to those of proliferating hepatocytes after PHx, suggesting the recapitulation of regeneration *in vitro* (Hu et al., 2018). Following chronic hepatic or biliary injury, hepatocytes were found to undergo reprogramming into progenitor-like cells for regeneration, and the Hippo–Yap signaling appears to play an essential role (Li et al., 2019). Indeed, YAP activation in organoids derived from perfused hepatocytes induced the expression of endogenous liver progenitor genes and exhibited a strong clonogenic capacity, supporting that the Hippo signaling may control the liver cell fate (Yimlamai et al., 2014). Adult resident cholangiocytes may also regenerate the liver after chronic damage (Raven et al., 2017; Deng et al., 2018; Manco et al., 2019; Russell et al., 2019). KRT19^+^ cholangiocyte organoids were shown to convert to hepatocytes, with expression of albumin but not KRT19, upon transplantation into CCl_4_–retrorsine-induced acute liver damage (Huch et al., 2015). By comparing the transcriptome and epigenetic regulators of cholangiocyte organoids with cholangiocytes in hepatotoxin 3,5-diethoxycarbonyl-1,4-dihydro-collidin (DDC)-injured livers, similar genome-wide changes were identified and were licensed by TET1-mediated hydroxymethylation, which further supported that liver organoid is a model in studying tissue regeneration upon liver injuries (Aloia et al., 2019).

Liver organoids also have the potential to mimic hepatocyte regeneration during the liver homeostasis. A recent study proposed that Wnt-responsive cells by Axin2 labelling acted as progenitors during physiological conditions (Wang et al., 2015). By isolating Lgr5^+^ Wnt-responsive cells in CCl_4_-damaged livers, it was found Lgr5^+^ cells expand extensively in an organoid culture (Huch et al., 2013). The clonal organoids expressed multiple hepatocyte-lineage markers, suggesting a possible population of Wnt-responsive progenitors in the liver. The progenitor role of Axin2^+^ or Lgr5^+^ cells however remained controversial, as other independent experiments showed a limited contribution of these cells in liver regeneration (Planas-Paz et al., 2016; Ang et al., 2019; Chen et al., 2020; Sun et al., 2020). Nevertheless, liver organoids offer a convenient way to look into the regeneration process and may provide insights to manipulate the regeneration process.

### Modeling liver cancers

Liver cancer is the sixth most common cancer and forth most malignancy worldwide (Bray et al., 2018). Late diagnosis and tumor heterogeneity contribute to high mortality of liver cancer patients (Marquardt et al., 2015). Genetic engineering of hepatocyte or cholangiocyte organoids with potential driver oncogenes provides a tractable and feasible system to model liver cancer initiation. It is also possible to study the structural and functional changes during the carcinogenesis in engineered organoids.

By the introduction of c-MYC, an important oncogene in human hepatocellular carcinomas (HCCs), into the organoids derived from transdifferentiated hepatocytes, Sun et al. (2020) observed excessive contacts between the mitochondria and the endoplasmic reticulum membranes, which appeared to be an unrecognized oncogenic event in liver carcinogenesis (Sun et al., 2019). Moreover, by tracing the process of RAS-induced carcinogenesis in hepatocyte organoids, hepatocytes gradually expressed cholangiocyte markers, such as CK7, CK19, SOX9, and SPP1, and began to possess intracellular cavities filled with microvilli, a hallmark ultrastructure of intrahepatic cholangiocarcinoma (ICC) cells (Sun et al., 2019). It was the first documentation of the conversion of human hepatocytes into ICC cells, therefore supporting that hepatocytes could be the cell of origin for ICC. Other common cancer genes have also been analyzed in hepatocyte and cholangiocyte organoids. Mutations affecting the proposed epigenetic regulator BAP1, a deubiquitinating enzyme, are involved in ICCs (Jiao et al., 2013). BAP1 loss of function in human cholangiocyte organoids disrupted epithelium organization and cell polarity. Genomic analyses showed that BAP1 deficiency in human cholangiocyte organoids affected chromatin accessibility related to junctional and cytoskeletal genes, which appeared to be critical for the acquisition of malignant features in human ICCs (Table 2; Artegiani et al., 2019).

**Table 2:** **Modeling liver diseases with organoids**.

**Type of diseases**	**Name of diseases**	**Features of diseases**	**Contents of liver organoids**	**References**
Liver cancer	HCC	PDOs	Needle biopsies	Nuciforo et al., 2018
			Tumor specimens	Broutier et al., 2017
		HCC with c-MYC mutation	Reprogrammed hepatocyte-derived organoids	Sun et al., 2019
	CC	PODs	Needle biopsies	Nuciforo et al., 2018
			Tumor specimens	Broutier et al., 2017
		ICC with RAS mutation	Reprogrammed hepatocyte-derived organoids	Sun et al., 2019
		CC with BAP1 mutation	Primary cholangiocytes	Artegiani et al., 2019
	CHC	PDOs	Tumor specimens	Broutier et al., 2017
Chronic liver diseases	Virus infection	HBV infection	PH-derived organoids	Fu et al., 2019
			Vascularized liver organoids (PSC-derived)	Nie et al., 2018
	Fatty liver disease	Steatohepatitis	PSC-derived liver organoids (hepatocytes, cholangiocytes, stellate cells, and Kupffer-like cells)	Ouchi et al., 2019
		Alcoholic fatty liver	PSC-derived liver organoids + fetal liver MSCs	Wang et al., 2019
	Fibrosis	Drug-induced fibrosis	HSCs + HepaRG	Coll et al., 2018; Leite et al., 2016
Developmental liver diseases	Genetic disorders	A1AT deficiency	Patient-derived cholangiocyte organoids	Huch et al., 2015
		ALGS	PSC-derived hepatobiliary organoids	Guan et al., 2017
		Cystic fibrosis with CFTR mutation	PSC-derived cholangiocyte organoids	Ogawa et al., 2015; Sampaziotis et al., 2015

CC, cholangiocarcinoma; CHC, combined hepatocellular cholangiocarcinoma.

**Figure 3:**
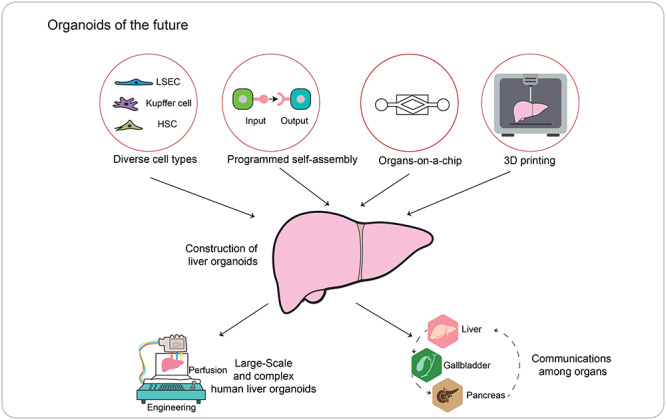
Future potentials of liver organoids. The future effort will focus on taking advantages of diverse technologies, including bioengineering, programmed self-assembly, etc. to assemble diverse types of liver cells into organized and complex liver organoids. We could imagine the construction of liver unit of large scale or even acquisition of diverse functional organ units for studying communications among organs.

PDOs are another useful systems for cancer studies. They recapitulate features of primary cancers and enable analyses of cancers at the malignant stage. PDOs of liver cancers have been established by a few groups even using needle-punctured samples. PDOs of liver cancers were mainly derived from poorly differentiated tumors, with the overall efficiency of 37.5% for resected specimen and 26% for biopsies samples (Broutier et al., 2017; Nuciforo et al., 2018). Liver cancer PDOs demonstrated the histological architecture and genomic landscape of primary liver cancers and, importantly, were able to reflect the intratumoral heterogeneity (Nuciforo et al., 2018). In that way, PDOs allowed the identification of patient-specific drug sensitivities and the establishment of a platform to inform drug development. Upon tested *in vitro*, liver cancer PDOs showed the ability to present the different response to sorafenib, a drug with limited responses in HCC patients. Moreover, drug screening in liver cancer PDOs revealed sensitivities to an ERK inhibitor SCH772984, implicating ERK as a potential target for liver cancer treatment (Table 2; Broutier et al., 2017).

### Modeling other liver diseases

Other chronic liver diseases were modeled by liver organoids, including hepatitis B virus (HBV) infection, steatosis, and fibrosis, all of which are common and lack an appropriate therapy. 3D-differentiated hepatocyte organoids derived from proliferated PHs or liver specimens have been used in HBV-host infections (Fu et al., 2019). NTCP, a HBV entry receptor, was expressed after forming hepatocyte organoids. After HBV infection, these hepatocyte organoids produced the main HBV transcript of 3.5 kb and viral transcription template cccDNA, suggesting HBV replication in hepatocyte organoids (Fu et al., 2019). Complex liver organoids generated by self-assembly of PSC-derived hepatocytes, MSCs, and HUVECs acted as another model for HBV studies (Nie et al., 2018). NTCP was expressed at an even higher level in organoids after the coculture with MSCs and HUVECs. Moreover, this complex liver organoid could maintain long-term propagation of HBV accompanied with hepatic dysfunction and altered hepatic ultrastructure (Table 2; Nie et al., 2018).

Risk factors of many chronic liver diseases affect both hepatocytes and other types of cells. Complex organoids are developed to model the complex cell–cell interactions in steatohepatitis. Human PSCs were differentiated to organoids containing hepatocyte-like cells, HSCs, cholangiocytes, and Kupffer cells. Remarkably, upon free fatty acid treatment, such liver organoids became steatotic, fibrotic, and infiltrated with immune cells, which were key features of patients with steatohepatitis (Ouchi et al., 2019). Liver organoids derived from coculture of hepaRG cells and HSCs were used for the assessment of fibrogenesis. Exposure of organoids to TGFβ and thioacetamide induced the expression of fibrosis marker and the secretion of pro-collagen type I. These organoids also showed response to hepatocyte-mediated toxicity, indicating an appropriate model to study drug-induced fibrosis (Leite et al., 2016; Coll et al., 2018). Others have cocultured PSC-derived liver organoids with MSCs, and these organoids responded to ethanol and showed pathologic changes associated with alcoholic liver diseases (Table 2; Wang et al., 2019).

There are studies exploiting patient-derived hepatocytes and cholangiocytes to generate organoids in modeling genetic diseases. To that end, organoids from patients with A1AT deficiency (Huch et al., 2015), ALGS (Guan et al., 2017), cystic fibrosis (Ogawa et al., 2015; Sampaziotis et al., 2015), and Wolman disease (Ouchi et al., 2019) have been reported to recapitulate the development of these diseases and serve as a platform to study drug responses (Table 2).

### Translational studies for regenerative medicine

Liver transplantation is the only effective treatment for patients with end-stage liver diseases. However, lack of proper donors limits the application of liver transplantation. One goal of organoid application is to serve as an alternative solution for liver organs in transplantation. It has been shown that fetal PH-derived organoids engrafted and repopulated injured livers in the fumarylacetoacetate hydrolase (FAH)-deficient mice, a model for fatal metabolic liver disease (Hu et al., 2018). Notably, organoid graft grew rapidly after 1 month, and repopulated cells continued to express hepatic markers for an extended period of 2–3 months. Meanwhile, cells in organoid grafts became further mature and barely expressed fetal hepatocyte marker AFP (Hu et al., 2018). Vascularized liver buds were also transplanted to liver failure mice. Indeed, transplantation of liver buds containing PSC-derived endoderm hepatic cells, stromal cells, and endothelial cells improved the survival of TK-NOG mice, which developed liver failure after treatment with ganciclovir (Takebe et al., 2013). Higher levels of human albumin secretion were detected in mice serum, as well as improved capacity of the human-specific metabolism of diclofenac (Takebe et al., 2017).

Massive production of liver organoids with reproducibility would shed light on their usage in clinics. Cryopreserved human PHs could be expanded up to 10000-fold after several passages, which are transplantable and could be further developed for translational studies (Zhang et al., 2018). LGR5^+^ human cells could go through large-scale expression and differentiation in spinner flasks. After 6 weeks, a total amount of 10^10^–10^12^ cells was acquired with a starting number of 10^6^ cells (Schneeberger et al., 2020). Expansion of human PSC-derived vascularized liver buds in Omni-well array culture platform provides a way for getting >10^8^ organoids with reproducible hepatic functions and therapeutic advantages in treating animals with liver failure (Takebe et al., 2017). Indeed, in order to accelerate the organoid application in clinical translation, a center for vascularized liver bud transplantation initiative is now being established in Japan for studies on large animals and treatment of pediatric metabolic liver diseases (Takebe et al., 2018).

## Perspectives

Liver organoids provide a useful tool for studies on liver development and regeneration. Thanks to the inclusion of cells and living environments, liver organoids could also be widely applied for modeling chronic liver diseases, hepatitis, biliary tract diseases, metabolic diseases, vascular disorders, and liver cancers to understand the underlying pathological mechanisms and find potential treatments. Besides modeling adult liver disorders, future endeavors will enable the generation of fetal hepatocyte organoids or hepatoblastoma organoids for looking into pediatric liver diseases, such as biliary atresia, ALGS, Wilson disease, and, specifically, hepatoblastoma, the most common cancer of fetal livers.

In particular, because of its specific metabolism ability, liver organoids hold great promise for drug testing in the clinic. Biobanks of human liver cell lines and organoids have been established and would be enlarged with high expectations for testing efficacy and toxicity of drugs in clinical studies and personalized medicine, as they harbored the possibility to reflect features of human liver tissues, including basic functions and certain heterogeneities (Qiu et al., 2019). Alternatively, engineered liver cancer organoids could be further applied in modeling and studying liver cancer initiation from additional perspectives for preventive therapy. With the advantages of capturing molecular and structural aberrances by introduced oncogenes, it represents a tractable *in vitro* model for understanding the oncogenic processes during human cancer development. Besides driver genes, the cancer microenvironment cells play important roles during liver cancer progression and metastasis. It is possible and equally significant to include other types of cells, such as stromal components and immune cells in the future for analyzing cellular interactions and signaling cross talks during carcinogenesis (Tuveson and Clevers, 2019). Moreover, it would be a plausible human model to study hormones in sex-related differences of liver cancer development, which is represented with a marked male predominance (Li et al., 2012).

Despite the simplicity and manipulability, current liver organoids are still somewhat different from the complex structures and functions of their *in vivo* counterparts. Positional cell contacts and regional signaling transductions offered by organized liver structure are also responsible for better modeling liver development, regeneration, and diseases. Generating organoids with complex and organized structures is the upmost goal in the field, not limited to the liver. Along with other related techniques, it is possible to generate complex organoids of multiple types of tissues. Recent progress in genome scale analyses focusing on gene expressions and cell interactions greatly enhanced the understanding of *in vivo* organ development, which will apparently benefit the development of complex organoids *in vitro* by self-organization (MacParland et al., 2018; Aizarani et al., 2019). Indeed, hepatobiliary–pancreatic organoids have been recently developed (Koike et al., 2019).

Though self-assembly of differentiated cells has enabled the development of complex liver organoids, such process appeared to be spontaneous and not fully controllable. Improved engineering, enabling a precise control of the nutrition and cell location, may facilitate the fidelity of organoid construction. Creating a signaling center by genetic engineering a part of cells has been shown to induce signaling gradients in brain organoids (Cederquist et al., 2019), which may help to specify the positional identity of cells in liver organoids. Synthetic elements, such as Notch and its ligands, could also be designed and introduced to organoids to deliver specific cell–cell contact for programming the customized self-assembly (Toda et al., 2018). In addition to these tools governing cellular signalings, organoid chips provided precise control of positional signals and niche environment. The design principle of organoid chips is to prepare different groups of cells with specific distribution instructions and multiple cultural conditions similar to the *in vivo* microenvironment, therefore guiding the formation of proper structures (Park et al., 2019).

Currently, liver organoids are ∼100–500 μm in size, which are much smaller than the size of a real organ. Human livers are at the centimeter scale, with perfusable vascular and biliary systems. Increasing the scale of liver organoids would be needed for multiple applications. Fabrication of a scaffold that supports the ordered assembly of vessel network and hepatic cells surrounding a perfusable microchannel network provided an idea for scalable production of millimeter-sized tissues (Zhang et al., 2016). Perfusable vascular system *in vitro* may facilitate the oxygen and nutrition infiltration when the size of liver organoids is >200 μm. In that way, the oxygen and nutrition would flow into an open capillary in liver organoids and connect with outside vessels. Recently, sustained perfusion of revascularized liver scaffolds has been achieved by incorporating HUVECs into decellularized porcine livers (Shaheen et al., 2020). As looking forward, we would expect transplantable livers at the centimeter scale with direct surgical anastomosis to patient vessels and establishing immediate blood perfusion (Figure 3).
